# Impact of urbanization and gardening practices on common butterfly communities in France

**DOI:** 10.1002/ece3.2526

**Published:** 2016-10-18

**Authors:** Benoît Fontaine, Benjamin Bergerot, Isabelle Le Viol, Romain Julliard

**Affiliations:** ^1^ Département Ecologie et Gestion de la BiodiversitéMuséum National d'Histoire NaturelleUMR 7204 – Conservation des espèces, suivi et restauration des populations, Centre d'Ecologie et des Sciences de la ConservationParisFrance; ^2^ UMR CNRS 6553 ECOBIO – Université de Rennes 1ParisFrance

**Keywords:** gardening practices, Lepidoptera, monitoring, refuge, urbanization impact

## Abstract

We investigated the interacting impacts of urban landscape and gardening practices on the species richness and total abundance of communities of common butterfly communities across France, using data from a nationwide monitoring scheme. We show that urbanization has a strong negative impact on butterfly richness and abundance but that at a local scale, such impact could be mitigated by gardening practices favoring nectar offer. We found few interactions among these landscape and local scale effects, indicating that butterfly‐friendly gardening practices are efficient whatever the level of surrounding urbanization. We further highlight that species being the most negatively affected by urbanization are the most sensitive to gardening practices: Garden management can thus partly counterbalance the deleterious effect of urbanization for butterfly communities. This holds a strong message for park managers and private gardeners, as gardens may act as potential refuge for butterflies when the overall landscape is largely unsuitable.

## 1 Introduction

Study of cross‐scale interactions on patterns of biodiversity is of growing concern in ecological studies and aims at understanding how fine‐scale processes can influence a broad spatial extent, or, conversely, how broadscale drivers impact fine‐scale dynamics (see a review in Peters, Bestelmeyer, & Turner, 2007). Analyses of the effects of landscape context and local conditions (organic/conventional farming, habitat patch size, local vegetation type) on species diversity or abundance (e.g., Cornell & Donovan, 2010; Roschewitz, Gabriel, Tscharntke, & Thies, 2005; Vergara & Armesto, 2009) show that species dynamics are influenced by interactions across spatial scales. However, the effects of these interactions are complex, and the relative importance of local vesus landscape factors depends on taxa, for instance with the level of habitat specialization (Pandit, Kolasa, & Cottenie, 2009) or with dispersal abilities (Schmidt, Thies, Nentwig, & Tscharntke, 2008).

Butterflies are a valuable model for such studies, because of their importance in ecosystems as plant pollinators (Ehrlich, 2003) and prey for other organisms (e.g., Murakami & Nakano, 2000; Strong, Sherry, & Holmes, 2000), and because their short life cycle and contrasted dispersal abilities make them good models to study the impact of environmental variables. These characteristics are shared with other invertebrates, but butterflies are comparatively much better known than other invertebrate taxa (New, 1997), which allow for a large range of ecological studies. Indeed, for butterflies, it has been shown that species diversity and abundance is influenced by landscape complexity and type of farming (Rundlöf & Smith, 2006), quality of habitat (Pocewicz, Morgan, & Eigenbrode, 2009) or habitat management (Marini, Fontana, Battisti, & Gaston, 2009). Last but not least, butterflies in Europe are well covered by many field guides, which makes them easy to identify, a main prerequisite for using data from nonspecialists.

Urbanization is known to deeply impact biodiversity patterns (Bergerot, Fontaine, Julliard, & Baguette, 2011; Garaffa, Filloy, & Bellocq, 2009; Knapp, Kuhn, Mosbrugger, & Klotz, 2008; Magura, Lovei, & Tothmeresz, 2008; Muratet et al., 2008): As such, the importance of studies and conservation actions across several scales in urban context has been emphasized (Savard, Clergeau, & Mennechez, 2000). Representing green oases in an inhospitable matrix, gardens are recognized as potentially important resource for butterflies in anthropogenic environment, especially as a food source for adults (Toms, Humphreys, & Kirkland, 2010; Vickery, 1995). Di Mauro, Dietz, and Rockwood (2007) have studied at a medium scale (135 gardens surveyed over ca. 10,000 km²) the interacting effects of urbanization and gardens on butterfly populations and have shown that butterfly diversity is negatively affected by urbanization, but that the urban matrix is just one factor determining species diversity.

In the present study, we further investigated the interacting impacts of urbanization and local garden characteristics on the total abundance and richness of butterfly communities in France. We used a long‐term dataset from a nationwide citizen science monitoring program that allows testing for various garden characteristics and identifying species‐specific responses. Our aim was to identify the gardening practices the most beneficial for the mitigation of urban impact on butterfly communities.

## 2 Methods

### 2.1 Sampling protocol

#### 2.1.1 Garden butterfly monitoring scheme protocol

This study is based on data collected in the framework of the French Garden Butterfly Monitoring Scheme (Observatoire des Papillons des Jardins—OPJ—http://vigienature.mnhn.fr/page/biodiversite-des-jardins), a nationwide butterfly monitoring scheme open to the general public. Participants identify and count butterflies in their gardens, from a closed list of 28 common species or species groups. Among these Lepidoptera species is one common diurnal moth, *Macroglossum stellatarum*, often found in gardens. For the sake of simplicity, in the text below, the 28 monitored species/species groups are referred to by the term “butterflies”, even if they include *M. stellatarum*. Seven of the 28 monitored species/species groups have specific host plants and can be qualified as specialist species: *Aglais urticae*,* Argynnis paphia*,* Cacyreus marshalli*,* Inachis io*,* Vanessa atalanta* and *Limenitis* spp. (Lafranchis, Jutzeler, Guillosson, Kan, & Kan, 2015). For each species/species group, monthly figures provided by participants represent the maximum number of butterflies seen simultaneously. Counting takes place from March to October. Localization information are restricted to the municipality (smallest administrative district in France) to which the garden belongs. In addition, the observer is prompted to fill a short questionnaire on the landscape around the garden and on the garden itself: garden area, presence of garden features such as lawn, pond, orchard, fallow, use of pesticides, type of plants from a closed list.

### 2.2 Analyses

Using the OPJ data for the years 2006 to 2012, the average monthly abundance was calculated for each species/species group in each of the 10,619 participating gardens. In order to reduce the problem of heterogeneity in the dataset due to nonindependence between individual detection probability for species seen in groups, all monthly abundances which were above 10 (0.4% of all data) were levelled to a maximum value of 10 (Julliard, Clavel, Devictor, Jiguet, & Couvet, 2006). For each garden, average monthly species richness, average monthly total abundance (all species pooled together) and average monthly abundance of each species/species group were tested against several variables.

These variables were as follows:

#### 2.2.1 Landscape variables

1. Urbanization: Proportion of artificial area (i.e., buildings, infrastructures) in the municipality, as given by the first level of Corine Land Cover 2000 (Artificial surfaces, EIONET, 2009).
2. Natural habitats: Proportion of natural and seminatural area (i.e., forests, shrublands, natural grasslands, as opposed to farmland) in the nonartificial area in the municipality, as given by the first level of Corine Land Cover 2000 (Forest and semi‐natural areas, EIONET, 2009).

#### 2.2.2 Local variables

1. Garden area;
2. Naturalness: Index of naturalness of the garden: In the garden description, fallow, nettles *Urtica dioica*, ivy *Hedera helix*, and brambles *Rubus fruticosus* are scored one if present, zero if absent. For each garden, the naturalness index was calculated as the sum of these scores;
3. Nectar: Index of nectar offer of the garden: In the garden description, the presence of *Buddleia*, knapweed (*Centaurea* spp.), lavender (*Lavandula* spp.), bramble is scored three; it is scored two for valerian (*Valeriana* spp.), clover (*Trifolium* spp.) and aromatic plants, and one for *Pelargonium*. These values were extracted from Bergerot, Fontaine, Renard, Cadi, and Julliard (2010), where plant species have been ranked according to their attractiveness for butterflies. For each garden, the nectar reward index was calculated as the sum of these scores.
4. Pesticides: In the garden description, the use of pesticides (insecticides, herbicides, fungicides, snail pellets, and/or Bordeaux mixture) is included. It was scored 0 if no pesticide use was reported, 1 otherwise.

Index of naturalness and index of nectar offer are considered independent from each other (*R*² = .31).

Impact of the presence of three host plants (i.e., nettles, ornamental *Pelargonium,* Brassicaceae) was also tested. It was tested separately from the effect of naturalness and nectar reward, as they were not independent (e.g., *R*² = .57 between the presence of nettles and garden naturalness, *R*² = .54 between presence of fallow and garden naturalness).

In order to assess the impact of these explanatory variables on the average species richness and the average total abundance of butterfly communities, type III ANOVAs (*F*‐test) were computed on generalized linear models with second‐order interactions among the explanatory variables. We assumed a quasi‐Poisson distribution to correct for overdispersion. In order to account for spatial autocorrelation, second‐degree polynomial terms of the spatial coordinates of the sample locations (latitude and longitude of the centroid of the garden district) were included in the model (Lichstein, Simons, Shriner, & Franzreb, 2002). As municipality areas vary across France, area of the garden municipality was also included in the model.

To investigate how species specific were the results on the community richness and total abundance, we run a similar model on the mean abundance per month of each species. We tested the correlation between the slope estimates of the effects of urbanization and those of garden naturalness and nectar offer effects with a Pearson test.

All statistical calculations were made with R statistical software (R Development Core Team, 2009), with the CAR package.

## 3 Results

### 3.1 Landscape variables

**Figure 1:**
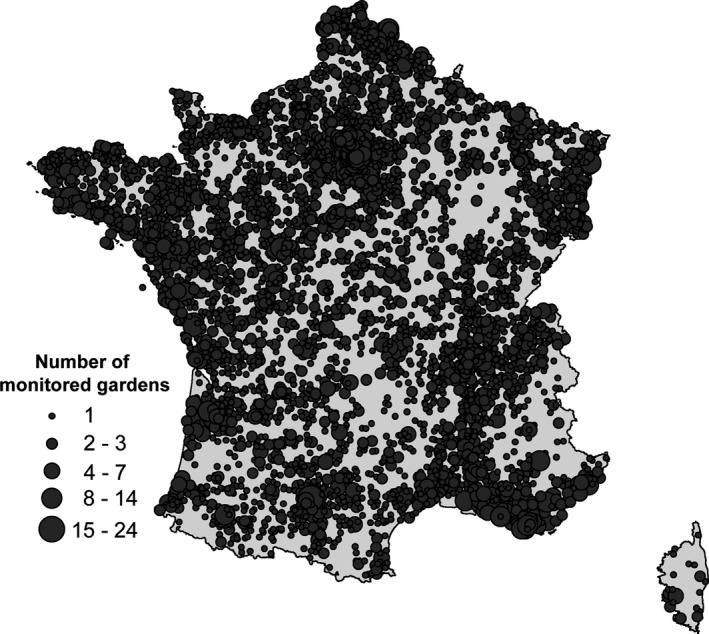
Monitored gardens in France between 2006 and 2012 in the framework of the Observatoire des Papillons des Jardins (Garden Butterfly Observatory—OPJ)

Between 2,100 and 4,000 gardens were monitored each year (Figure 1). This represents 100,563 monthly surveys and ca. 1,300,000 butterflies counted. Average monthly butterfly species richness and total abundance were significantly negatively correlated with urbanization (Table 1). Among the 28 surveyed species/species groups, the abundance of 15 was significantly negatively correlated with the amount of urbanization in the municipality (Table S1).

**Table 1:** Results of the model on average species richness and abundance (all species) of butterflies monitored in gardens in France, 2006–2012

	Average species richness	Average total abundance
Garden area	***>	***>
Garden naturalness		
Garden nectar offer		***>
Pesticides use	***<	***<
Nettles	***>	***>
Pelargonium	***<	
Brassicaceae	***>	***>
Urbanization	***<	***<
Natural habitat		
Area × naturalness		
Area × nectar	**<	*<
Area × pesticides		
Urbanization × area		*>
Natural habitat × area	*<	
Urbanization × Naturalness		
Urbanization × Nectar offer		
Urbanization × pesticides		
Natural habitat × Naturalness	*>	
Natural habitat × Nectar offer		
Natural habitat × Pesticides		

“<” and “>” denote negative and positive effects, respectively, and asterisks, the associated *p*‐value, *p < .05, **p < .01; ***p < .001.

Although the amount of natural habitat in the municipality did not have an effect on community abundance or richness, it had positive or negative impact on four and three species/species groups, respectively.

### 3.2 Local variables

**Figure 2:**
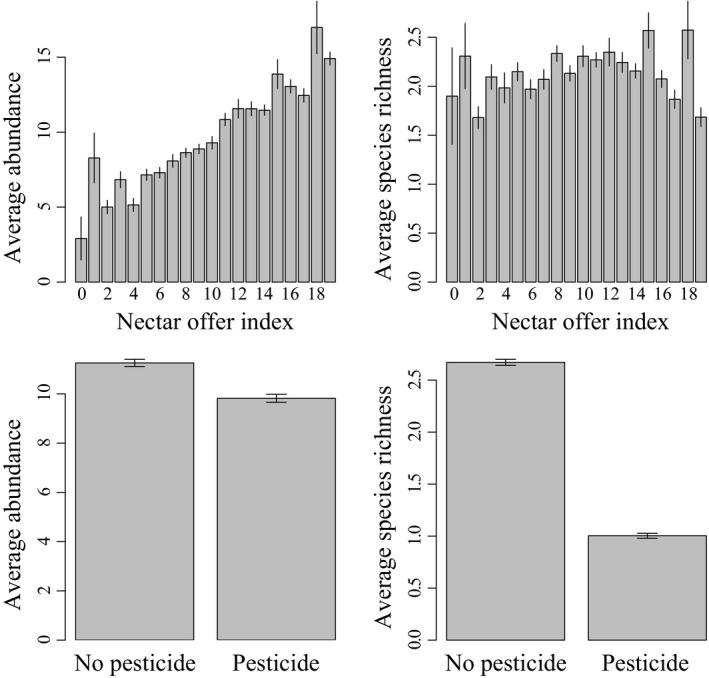
Effect of nectar offer index and pesticide use on average monthly abundance and species richness in monitored gardens in France, 2006–2012

Average monthly species richness and total abundance of butterfly communities were significantly positively correlated with garden size, as was the abundance of 22 species/species groups. Average monthly abundance was significantly positively correlated with nectar offer in the garden. This positive effect was significant for 19 of the species/species groups. Both the average total abundance and richness of butterfly communities were negatively correlated with pesticide use (Table 1 and Figure 2). This negative effect was detected for eight of the species/species groups (Table S1).

Although the garden naturalness did not have a significant effect on community abundance or richness, it had a significant positive or negative impact on five and six species/species groups, respectively.

We found a significant negative interaction between garden size and garden nectar offer on both the species richness and the total abundance of butterfly communities: The positive effect of a large nectar offer is greater in large gardens.

The abundance of 16 species/species groups (including those having nettles as obligatory host plant) was significantly positively correlated with the presence of nettles in gardens. The abundance of four species/species groups (including *Cacyreus marshalli*, whose host plants are *Pelargonium*) was positively correlated with the presence of ornamental *Pelargonium* in gardens, whereas three others were negatively impacted. The abundance of 16 species/species groups was significantly positively impacted by the presence of Brassicaceae in gardens, including White Pieridae and *Anthocharis* spp. which use these plants as host plants (Table 1).

### 3.3 Interactions between landscape and local variables

We found a significant positive interaction between the level of urbanization and garden area on the average abundance of butterfly communities, indicating that the positive effect of garden area become stronger in highly urbanized landscapes. This interaction was also found for four of the species/species groups.

We found a significant negative interaction between the amount of natural habitat in the surrounding landscape and the garden area on butterfly species richness. This indicates that the benefit of having large garden is weaker when the surrounding landscape includes a lot of natural habitats. This interaction was also found for five of the species/species groups.

Finally, we found a positive interaction between the amount of natural habitat in the surrounding landscape and the garden naturalness on butterfly species richness, indicating that the positive impact of garden naturalness is stronger when the garden is surrounded by natural habitats. However, this interaction was significant only for two species/species groups.

### 3.4 Species‐specific response

All specialist species except *A. paphia* were positively impacted by nectar offer in the garden.

**Figure 3:**
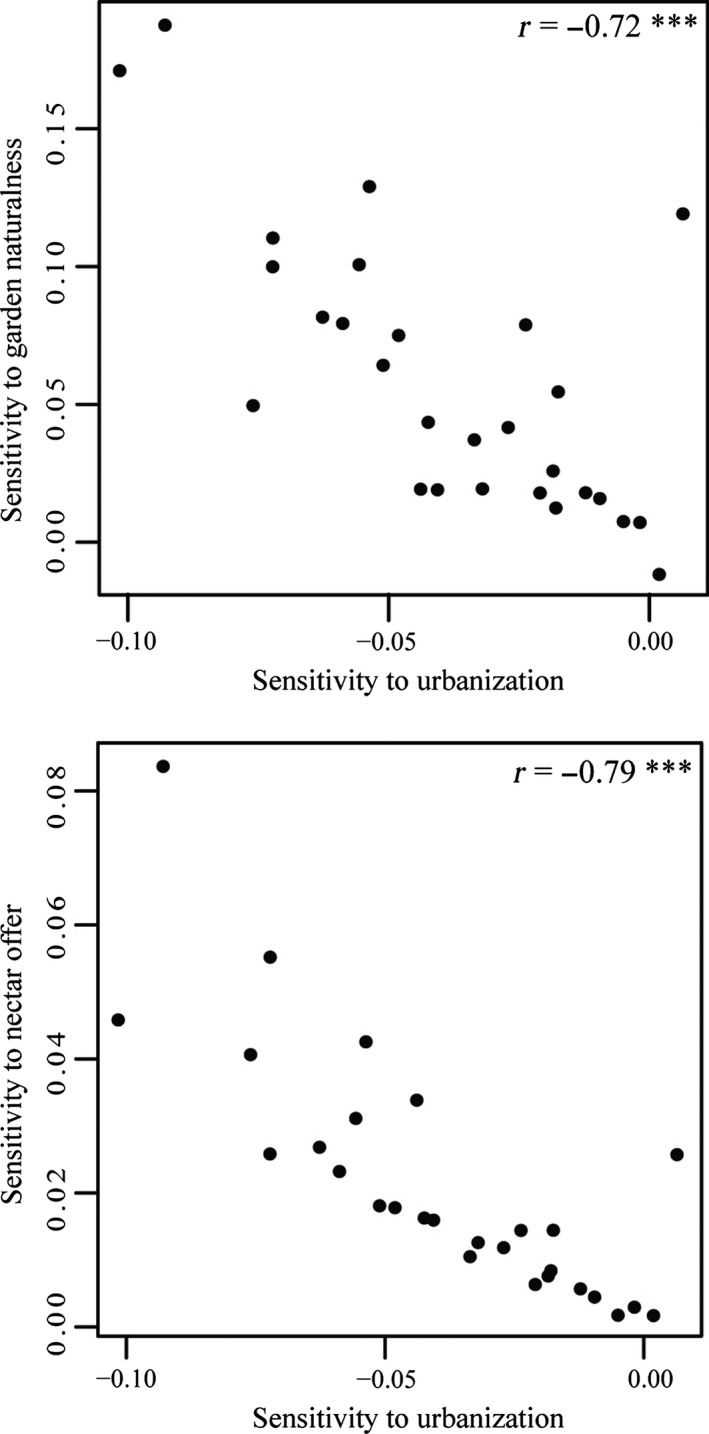
Relationships between slope of response to urbanization and slope of response to garden naturalness (left) and nectar offer in the monitored gardens in France, 2006–2012

Across all the species/species groups monitored, the response to urbanization and the response to garden naturalness were significantly negatively correlated (corr. coef = −.72, *t* = −5.42, *p* < .001). Similarly, the response to urbanization and the response to nectar offer were significantly negatively correlated (corr. coef. = −.79, *t* = −6.57, *p* < .001): Species having the steeper negative response to urbanization (urban avoiders) are those that benefit the most from the garden characteristics (Figure 3).

## 4 Discussion

We confirmed that at the landscape scale, urbanization has a negative impact on butterfly abundance and species richness. Such deleterious effects of urbanization have already been shown (Bergerot et al., 2011; Di Mauro et al., 2007). However, our large‐scale study demonstrates that this negative impact can partly be mitigated at a local scale by garden characteristics and gardening practices, such as the nectar offer and the absence of pesticide use: Butterfly‐friendly practices are efficient even in highly urbanized landscape and/or in small garden. Gardens represent wild species refugia in urban areas, and species that suffer the most from urbanization are the ones that benefit the most from garden naturalness and nectar offer. Urban avoider butterfly species, that is, species which are too specialized to cope with urban environment, were the more sensitive to local variables, garden characteristics, and gardening practices in particular. This is in accordance with Pandit et al. (2009) prediction that habitat specialists respond primarily to local factors, compared with habitat generalists which respond primarily to regional spatial processes. However, Lizée, Mauffrey, Tatoni, and Deschamps‐Cottin (2011) have demonstrated that fragmentation is the first factor affecting butterfly communities, before local management. This may be related to the various dispersal abilities of species, which is a primary factor explaining their presence or absence in town parks (Kozlov, 1996). Species ecology is also of great importance: Generalist species tend to survive better in an urban ecosystem compared with specialist species (Lizée, Tatoni, & Deschamps‐Cottin, 2015). As a consequence, while local management has a strong effect on the local butterfly community, in urban environment these communities will always be different from the ones of more natural environments, as specialist species with poor dispersal ability will tend to be absent from urban habitats, whatever good butterfly‐friendly management there is. Nevertheless, we show that negative effects of urbanization may be mitigated, and our results are important for park managers and private gardeners in cities, who could be prompted to orient their gardening practices to be more butterfly‐friendly in an efficient way (Matteson & Langellotto, 2010).

Several studies have shown that gardens represent food sources for butterflies (Toms et al., 2010; Vickery, 1995). The strong positive effect of nectar offer index we found clearly supports these findings: Nectar offer probably determines the garden carrying capacity. Moreover, six of seven specialist species/species groups were positively impacted by nectar offer in the garden: Even species having specific host plant requirements benefit from the presence of nectar plant, confirming that private gardens are used for foraging by adult butterflies, regardless of their requirements in terms of larval host plants. This is corroborated by a separate analysis (B. Fontaine, unpublished results) based on data from the seven specialist species only: Results were similar to those obtained with all species together; that is, nectar offer has a positive impact on butterflies. However, further investigations on this issue should include more specialist species than our dataset. Garden naturalness had no significant impact on butterfly abundance and diversity. However, as the response to garden naturalness depends on butterfly species, the composition of butterfly communities in gardens will be influenced by this garden descriptor, with urban avoider butterflies benefiting the most of garden naturalness. This is a strong result as gardening practices have thus the potential to mitigate the biological homogenization in urban areas (Mc Kinney, 2006). We hypothesize that for a given urbanization level, a higher naturalness index will favor some butterflies because they may breed in the garden, as several potential host plant species may be present (e.g., Apiaceae for *Papilio machaon*; Poaceae for *Melanargia* spp, Orange Hesperiidae, *Lasiommata* spp., *Maniola jurtina*,* Coenonympha pamphilus*,* Brintesia circe*, and *Pararge aegeria*; thistles for *Vanessa cardui*; nettles for *Aglais urticae*,* Vanessa atalanta*,* Inachis io*, and *Polygonia c‐album*). This impact of private gardens on butterfly reproduction was supported by our population level analysis: Several butterfly species benefit significantly from the presence of their host plant in the garden (White Pieridae, *Anthocharis* spp., *Aglais urticae*,* Vanessa atalanta* for instance). However, the abundance of several species is correlated with the presence of nettles or Brassicaceae, although these are not their host plants. Such nonspecific responses could be explained either because these plants act as important food source for these butterflies, or because their presence in private gardens correlates with the presence of other plant species which are the true host plants: For instance, it has been shown that nettles increase diversity and abundance of invertebrates which do not necessarily breed on nettles (Gaston, Smith, Thompson, & Warren, 2005).

It should be noted that the use of species groups, which allow the collection of large amount of data by the general public, masks difference between their constituent species and may blur species‐specific responses. For example, the group “White Pieridae” will probably be dominated in gardens by *Pieris rapae* and *P. brassicae*, which live in a wide variety of habitats, including anthropized ones, whereas *P. ergane* and *Pontia callidice*, in the same group, have a much more restricted niche (Lafranchis et al., 2015). Similarly, blue Lycaenidae make a most heterogeneous group, even if the majority of its data probably refer to *Polyommatus icarus*.

### 4.1 The power of a biodiversity citizen‐basedmonitoring

Our results also highlight the power of a biodiversity monitoring scheme based on the general public. When large datasets are concerned, citizen science programs offer several advantages compared with traditional ones. First, by relying on particular type of observers, here gardeners monitoring butterfly in their garden, they allow access to potentially restricted areas. Indeed, although representing a large part of the green spaces in urban areas, private garden are hardly studied because of access restriction to private properties. Second, monitoring programs involving nonspecialists allow gathering data over large spatial and temporal scales that could not be done by specialist as there is not enough manpower, and even if there was, the cost would be prohibitive (Levrel et al., 2010). Finally, taking part in such a scheme involves awareness raising, and hopefully, changes in observers daily behavior toward environment (Couvet, Jiguet, Julliard, Levrel, & Teyssedre, 2008).
