# Supplementary material for: Impact of urbanization and gardening practices on common butterfly communities in France
Source: Ecol Evol. 2016 Oct 18;6(22):8174–80. doi: 10.1002/ece3.2526 (PMC5108268; doi:10.1002/ece3.2526)
Supplement: Supplementary file 1 [file ECE3-6-8174-s001.docx]

Table S1: Results of the model on the abundance of 28 species/species groups monitored in gardens in France, 2006-2012. “<” and “>” denote negative and positive effects, respectively, and asterisks the associated P-value, * p < 0.05, ** p < 0.01; *** p < 0.001.

|  | Garden area | Naturalness | Nectar offer | Pesticides | Nettles | Pelargonium | Brassicaceae | Urbanization | Natural habitat | area x naturalness | area x nectar | area x pesticides | Urbanization x area | Natural habitat x area | Urbanization x Naturalness | Urbanization x Nectar offer | Urbanization x pesticides | Natural habitat x Naturalness | Natural habitat x Nectar offer | Natural habitat x Pesticides |
| --- | --- | --- | --- | --- | --- | --- | --- | --- | --- | --- | --- | --- | --- | --- | --- | --- | --- | --- | --- | --- |
| *Aglais urticae* | **> |  | ***> | **< | ***> |  | *> | **< | ***< |  | **< |  |  |  |  |  |  |  |  |  |
| *Aporia crataegi* | *> | *< |  | **< | *> |  |  |  |  |  |  |  |  | **< |  |  |  | *> |  |  |
| *Argynnis paphia* | *> |  |  |  | ***> |  |  | *< | *< |  |  |  |  |  |  |  | *< |  |  |  |
| *Brintesia circe* | *> |  |  |  |  |  |  |  |  |  |  |  | *> |  |  |  |  |  |  |  |
| *Cacyreus marshalli* |  | *< | **> |  |  | ***> |  |  | *> |  |  |  |  |  |  |  |  |  |  |  |
| *Coenonympha pamphilus* | ***> |  | **> |  | ***> |  |  | ***< | **> |  |  |  | *> |  |  |  |  |  |  |  |
| *Inachis io* |  | *< | ***> | **< | ***> |  | ***> | ***< |  |  |  |  |  |  |  |  |  |  |  |  |
| *Iphiclides podalirius* | ***> |  | ***> |  |  |  | *> | *< | ***> | *< |  |  |  |  |  |  |  |  |  |  |
| *Macroglossum stellatarum* |  | ***< | ***> |  |  | ***> | ***> | *< |  |  |  |  |  |  |  |  |  |  |  |  |
| *Maniola jurtina* | ***> |  | **> |  | ***> |  | ***> | ***< |  |  |  |  |  |  |  |  |  |  |  |  |
| *Papilio machaon* | ***> | *< | ***> |  |  |  | ***> | ***< |  | *< |  |  | ***> | *< |  |  |  |  |  |  |
| *Pararge aegeria* | **> | ***> |  |  | **> | ***> | ***> |  |  |  |  |  | *> |  |  | *> |  |  |  |  |
| *Polygonia c-album* |  |  | ***> |  | ***> |  | ***> |  |  |  |  |  |  | *< |  |  |  |  |  |  |
| *Vanessa atalanta* | **> |  | ***> | ***< | ***> |  | ***> | *< |  |  |  |  |  |  |  |  |  |  |  |  |
| *Vanessa cardui* |  | ***< | ***> |  |  |  | **> | ***< |  |  |  |  |  |  |  |  |  |  |  |  |
| Blue Lycaenidae^1^ | ***> | ***> | *> |  | ***> | *< | ***> |  |  | **< |  |  |  |  | *< |  |  |  |  |  |
| *Gonepteryx* spp. | ***> | *> |  | *< | ***> |  |  |  |  |  |  |  |  |  | *< |  |  |  | *> |  |
| *Callophrys* spp. | ***> |  | *> |  |  |  |  |  |  | *< | *< |  |  |  |  |  |  | *> |  |  |
| *Limenitis* spp. | ***> |  | *> |  |  |  |  | *< | *> | **< | *< |  |  | ***< |  |  | *< |  |  |  |
| *Melanargia* spp. | **> | **> |  | **< | ***> | **< |  | *< | *< |  |  |  |  |  |  |  |  |  |  |  |
| Orange Hesperidae^2^ | ***> |  | ***> |  | ***> | *< | *> |  |  |  |  |  |  | **< |  |  |  |  |  |  |
| Orange Lycaenidae^3^ | ***> |  | ***> | **< |  |  |  |  |  |  | *< |  |  |  |  |  |  |  |  | **> |
| *Anthocharis* spp. | ***> | ***> |  |  | ***> |  | **> | **< |  |  |  |  |  |  |  | **> |  |  |  |  |
| *Pyronia* spp. | **> |  | ***> |  | ***> |  | ***> | ***< |  |  |  |  |  |  |  |  |  |  |  |  |
| Speckled Hesperidae^4^ | ***> |  | **> |  |  |  |  |  |  | **< |  |  |  |  |  |  |  |  |  |  |
| *Lasiommata* spp. | **> |  |  |  |  |  | *> |  |  | *< |  |  |  |  |  |  |  |  |  |  |
| White Pieridae^5^ | **> |  | ***> | **< | ***> | ***> | ***> | ***< |  |  |  |  | *> |  |  |  |  |  |  |  |
| *Colias* spp. |  |  |  |  |  |  |  |  |  |  |  |  |  |  | **< |  |  |  |  |  |

1: Genera *Agriades*, *Albulina*, *Aricia*, *Celastrina*, *Cupido*, *Cyaniris*, *Eumedonia*, *Everes*, *Glaucopsyche*, *Iolana*, *Maculinea*, *Plebejus*, *Polyommatus*, *Pseudophilotes*, *Scolitantides*, *Vacciniina*

2: Genera *Hesperia*, *Ochlodes*, *Thymelicus*

3: Genera *Heodes*, *Lycaena*

4: Genera *Carcharodus*, *Pyrgus*, *Spialia*, *Syrichtus*

5: Genera *Leptidea*, *Pieris*, *Pontia*
